# Identifying, reducing, and communicating uncertainty in community science: a focus on alien species

**DOI:** 10.1007/s10530-022-02858-8

**Published:** 2022-08-29

**Authors:** Anna F. Probert, Daniel Wegmann, Lara Volery, Tim Adriaens, Rigers Bakiu, Sandro Bertolino, Franz Essl, Eugenio Gervasini, Quentin Groom, Guillaume Latombe, Dragana Marisavljevic, John Mumford, Jan Pergl, Cristina Preda, Helen E. Roy, Riccardo Scalera, Heliana Teixeira, Elena Tricarico, Sonia Vanderhoeven, Sven Bacher

**Affiliations:** 1 Department of Biology, University of Fribourg, Chemin du Musée 15, 1700 Fribourg, Switzerland; 2 Research Institute for Nature and Forest (INBO), Herman Teirlinckgebouw, Havenlaan 88 bus 73, 1000 Brussels, Belgium; 3 Faculty of Agriculture and Environment, Department of Aquaculture and Fisheries, Agricultural University of Tirana, Koder-Kamez, Tirane, Albania; 4 Department of Life Sciences and Systems Biology, University of Turin, 10123 Turin, Italy; 5 Global Change, Macroecology-Group, Department of Botany and Biodiversity Research, University Vienna, Rennweg 14, 1030 Vienna, Austria; 6 Centre for Invasion Biology, Department of Botany and Zoology, Stellenbosch University, Stellenbosch, South Africa; 7 Joint Research Centre, European Commission, Ispra, Italy; 8 Meise Botanic Garden, Meise, Belgium; 9 Institute of Ecology and Evolution, University of Edinburgh, Edinburgh, EH9 3JT UK; 10 Institute for Plant Protection and Environment, Belgrade, Serbia; 11 Centre for Environmental Policy, Imperial College London, Silwood Park Campus, Buckhurst Road, Ascot, SL5 7PY UK; 12 Institute of Botany, Czech Academy of Sciences, 252 43 Průhonice, Czech Republic; 13 Ovidius University of Constanta, Al. Universitatii nr.1, Corp B, 900470 Constanta, Romania; 14 UK Centre for Ecology and Hydrology, Benson Lane, Crowmarsh Gifford, OX10 8BB UK; 15 IUCN/SSC Invasive Species Specialist Group, Rome, Italy; 16 CESAM - Centre for Environmental and Marine Studies, Department of Biology, University of Aveiro, Campus Universitário de Santiago, 3810-193 Aveiro, Portugal; 17 Department of Biology, University of Florence, Sesto Fiorentino, FI Italy; 18 Belgian Biodiversity Platform - Département du Milieu Naturel et Agricole - Service Public de Wallonie, Avenue Maréchal Juin 23, 5030 Gembloux, Belgium

**Keywords:** Biodiversity monitoring, Citizen science, Data quality, Epistemic uncertainty, Linguistic uncertainty, Non-native species

## Abstract

Community science (also often referred to as citizen science) provides a unique opportunity to address questions beyond the scope of other research methods whilst simultaneously engaging communities in the scientific process. This leads to broad educational benefits, empowers people, and can increase public awareness of societally relevant issues such as the biodiversity crisis. As such, community science has become a favourable framework for researching alien species where data on the presence, absence, abundance, phenology, and impact of species is important in informing management decisions. However, uncertainties arising at different stages can limit the interpretation of data and lead to projects failing to achieve their intended outcomes. Focusing on alien species centered community science projects, we identified key research questions and the relevant uncertainties that arise during the process of developing the study design, for example, when collecting the data and during the statistical analyses. Additionally, we assessed uncertainties from a linguistic perspective, and how the communication stages among project coordinators, participants and other stakeholders can alter the way in which information may be interpreted. We discuss existing methods for reducing uncertainty and suggest further solutions to improve data reliability. Further, we make suggestions to reduce the uncertainties that emerge at each project step and provide guidance and recommendations that can be readily applied in practice. Reducing uncertainties is essential and necessary to strengthen the scientific and community outcomes of community science, which is of particular importance to ensure the success of projects aimed at detecting novel alien species and monitoring their dynamics across space and time.

**Supplementary Information:**

The online version contains supplementary material available at 10.1007/s10530-022-02858-8.

## Introduction

Community science—also sometimes termed ‘citizen science’ or ‘volunteer biological recording’—refers to the form of scientific inquiry involving public participation, usually through collaborative initiatives between volunteers and professional scientists (Jordan et al. 2015). For most community science projects, the aims are broadly twofold: to generate scientific data, whilst simultaneously engaging and educating community members about science and their environment. From a research perspective, public participation can benefit science, particularly in the stages of data collection and analysis, when practical caveats, such as lack of time, and economic or human resources, would represent a major constraint. This means that community science provides an alternative or complementary approach to address questions that otherwise would be logistically challenging or unfeasible under the common scientific framework (Dickinson et al. 2012; Newman et al. 2012; Pergl et al. 2020).

The scope and design of projects is usually determined by the primary objective, which may be more or less focused on generating scientific data or increasing education and community engagement, with the ultimate aim to lead to advances in both science and public understanding of science through a collaborative partnership between multiple sectors of society. The level of public participation can be considered a spectrum: whilst some projects are conceptualised and completed entirely by members of the public who may lack scientific backgrounds—such as community conservation groups (Peters et al. 2016)—others rely on a close partnership between community members and professional scientists, or alternatively, may be driven by professional scientists, involving the public only in the data collection stage (Haklay 2013; Pocock et al. 2015). These different models of project design, the respective levels of involvement of community members and experts, and the skills and experience of participants can result in varying types and degrees of uncertainty, which has led to questions about the reliability of community science datasets (Aceves‐Bueno et al. 2017).

Uncertainty is an inherent part of scientific research; however, the way it is identified, understood, and handled can strongly influence the degree to which data may be interpreted and used (van der Bles et al. 2019). It manifests due to limited knowledge—usually from incomplete information in the data collection stage and subsequent analysis of data—as well as through imprecise language at any stage of the research, where oral or written communication language may be interpreted differently among individuals. Uncertainties in community science projects are often overlooked, sometimes completely ignored, and previous attempts to provide solutions usually only consider them from an analytical perspective, concentrating on only certain types of uncertainty (e.g., measurement error) (Bird et al. 2014; Jiménez et al. 2019). Although the underlying uncertainties in natural conditions that arise using a community science approach do not differ from those under the common framework of science, they may vary in magnitude, either negatively or positively. For instance, as community science usually increases the “data collection power”, such projects may be expected to capture the natural variation of large-scale phenomena better than small-scale projects led by professional scientists (Baker et al. 2019). On the other hand, by favouring quantity, some community science projects have higher rates of measurement error or increased data collection bias compared to other approaches (Crall et al. 2011; Gardiner et al. 2012). Additionally, the bias and associated uncertainty resulting from poor experimental design is rarely quantified and considered in the analyses, although this is a problem that is not only associated with community science projects. Community science projects may underestimate the importance of setting a testable hypothesis and appropriate experimental design, which should consider trade-offs between data quality and data quantity; the design of community science projects is often a compromise between participation and data quality (Lewandowski and Specht 2015). These characteristics of community science can limit the conclusions that can be drawn from the data obtained or lead to its misinterpretation. Further, uncertainty is seldom properly communicated, leading to potential misunderstandings and mistrust among researchers, stakeholders and the public, and thus limits the use of data generated by community science projects in the decision-making process (Van der Bles et al 2019; Vanderhoeven et al. 2017).

Data generated by members of the public are now widely employed to monitor biodiversity and detect alien species in all environmental realms (Bois et al. 2011; Pearson et al. 2019; Perdikaris et al. 2017; Pusceddu et al. 2019; Stuart-Smith et al. 2017). This has been largely facilitated by the development of online tools, dedicated websites and smartphone applications that provide a simple and engaging way for community science participants to record their data (Adriaens et al. 2015; Giovos et al. 2019; Johnson et al. 2020; Rowley et al. 2019; Santori et al. 2021). In many cases, engaging community members in the data collection process may be the only practicable way to conduct large-scale or long-term studies or gain access to, and collect data in, difficult-to-access sites (Lepczyk 2005). As such, community science projects provide a practical tool for addressing invasion-related questions that require spatial, temporal, and/or phenological information (Roy et al. 2018), particularly in relation to post-border surveillance (Thomas et al. 2017). For instance, community science has been used to delimit the distribution of alien species, during both the early (Eritja et al. 2019; Hourston et al. 2015) and late (Bois et al. 2011; Crall et al. 2015) stages of invasion, to understand alien species’ range expansions (Grason et al. 2018), as well as temporal emergence patterns (Maistrello et al. 2016) and even to reveal impacts on native biodiversity (Mori et al. 2019; Roy et al. 2012). Given that community science projects often capture data opportunistically—particularly in the form of presence-only data—they may harbour large uncertainties that must be accounted for in downstream analyses (Bird et al. 2014; Isaac and Pocock 2015; Petersen et al. 2021). Additionally, the overall spatial and temporal data coverage from community science initiatives may be uneven; failing to account for this could make findings less robust for use in decision-making on the prevention and management of alien species.

The way uncertainty is communicated (or not) may affect trust in community science outcomes and ultimately decision-making that relies on community science data (van der Bles et al. 2019). Indeed, additional uncertainties may be introduced during the stage of communication. Many community science projects—though often implicitly—aim to achieve learning outcomes and increase the scientific literacy of their participants, which partly accounts for the engagement and motivation of volunteers (Jennett et al. 2016). The project design and methods need to be explained to participants for a thorough understanding of their role and to ensure these learning outcomes are achieved. This inherently includes communication on how specific aspects of project design, such as the way data are collected, relate to methodology to overcome biases and uncertainty. Furthermore, the endorsement of project results by other parties and societal actors equally requires open and transparent communication in order to reduce uncertainty, maintain the credibility of the research and uptake of community science projects within the decision-making process (Groom et al. 2019; van der Bles et al. 2019).

We argue that to produce scientifically robust and societal-relevant conclusions, projects must be carefully designed to identify and reduce potential sources of uncertainties. The analysis of the resulting data must adequately account for the remaining uncertainty, and the uncertainty associated with the findings must be effectively communicated.

Here, specific recommendations are provided to help increase the robustness of ongoing and future community science research projects and to increase the reliability of their research outcomes. Considering the importance of community science in alien species research, we focus on the application of community science to biological invasions in support of decision-making. We aimed to (1) outline four common research questions community science projects address when studying different aspects of alien species, then (2) identify the relevant sources of epistemic and linguistic uncertainty in the process of conducting a community science project, and (3) provide suggestions on how to reduce and account for epistemic uncertainties based on project aims. Finally, we aimed to (4) provide recommendations for effective communication explicitly addressing uncertainty towards participants, stakeholders, and end-users of project results.

The main concepts and ideas were developed during an interdisciplinary workshop, associated with the Alien CSI COST action working group, on the 23–24th September 2019 in Ljubljana, Slovenia, comprising of experts in community science, invasion science, social psychology, and statistics. The workshop was structured around the four topic sections of this paper (outlined above). Each topic was introduced by one presenter, then further developed in breakout groups of 4–6 individuals, followed by plenary discussions to collect and consolidate ideas, which then formed the basis for this work. This workshop was followed by a literature assembly and a consensus building process through the drafting of our work. To gain an overview of the current literature related to community science projects related to alien species, a search was conducted in the Scopus database in February 2020 using Publish or Perish version 7 (Harzing 2007) using the search string (“citizen science” OR “community science” OR “crowdsourcing science”) AND (“alien species” OR “exotic species” OR “introduced species” OR “non-native species” OR “biological invasions”). The resulting papers were screened and categorised as either relevant (i.e., papers that included any data derived from community scientists/volunteer recorders related to some ecological facet of alien species that were not review or overview papers) or irrelevant (i.e., papers that only used community science to assess native species or those that were related to accuracy or other human elements of community science). The remaining papers were then evaluated to identify key questions addressed and to determine whether expert verification of data was performed prior to analysis (see Supplementary material). Our search returned 194 papers, of which there was one duplicate entry and two inaccessible documents that were removed. Of the remaining, 83 were not considered relevant based on our criteria.

Here, we distinguish between professional scientists/experts and community scientists/volunteer recorders, adopting this terminology. However, we recognise that the expertise among participants of community science projects will vary greatly; some volunteer recorders may possess extensive knowledge relating to the study system and can indeed be considered experts that make more accurate and reliable observations, reducing data uncertainty. Further, whilst we acknowledge that volunteer recorders often play an important role in the management of alien species, we do not cover this here. Rather, we focus on the data collection and subsequent analysis and the communication of research findings.

### Project aims and key questions addressed in community science projects on alien species

Across the field of alien species research, we identified four key aims commonly addresses in the community science projects; these aims are largely driven by the need for information in decision-making on alien species policy and management. These were first identified during the workshop and then supported through our Scopus search (see Supplementary material).

To answer the research questions we identified, certain types of data must be generated e.g., presence data, abundance data, temporal data. Each of these can be subject to certain types of uncertainty, meaning that appropriate measures to reduce these should be considered. These are discussed further below.

(i) **Presence and distribution**: The first aim relates to verifying the presence of an alien species in a geographic area, addressing the question “does species A occur here”? This is relevant in terms of both species detection (i.e., detecting new incursions or confirming absence after a management intervention) and delimiting species distributions. Engaging community members in the surveillance of alien species means ‘many eyes on the ground’ and may facilitate early detection of novel species incursions (Ministry for Primary Industries 2016; Thomas et al. 2017), which can be a critical factor in eradication success (Vander Zanden et al. 2010; Wotton et al. 2004; but see Pluess et al. 2012). The early detection of alien species has been aided using smartphone and web applications allowing volunteer recorderss to submit species occurrences and obtain taxonomic verification in real-time (Moulin 2020). Additionally, delimiting the distributions of some alien species has only been possible due to public participants reporting sightings, which in certain cases may be more effective than common biological monitoring techniques (Goldstein et al. 2014).
(ii) **Abundance**: The second aim relates to evaluating the abundance of a specific alien taxon in areas in which it is known to occur. Estimates of abundance can be useful to understand the impact of alien species (Parker et al. 1999; Sofaer et al. 2018) and are important data to plan and evaluate management interventions. For harmful alien species for which eradication is no longer possible, maintaining populations below an ecological damage threshold might provide the most cost-effective management solution if feasible (Green et al. 2014; Robertson et al. 2020). Defining such thresholds requires some form of damage-density relationship and abundance data. Abundance estimates are therefore important to inform the management of alien species (Bradley et al. 2018). Measurements may be in terms of either the density (i.e., how many alien individuals are there in a given area?) or relative abundance (i.e., how many alien individuals are there in relation to native species of concern, e.g., parasites on a host or a plant). Information on relative abundance may also help understanding whether only vagrant/casual individuals are present, or whether an established population occurs. Community science projects that simultaneously assess the abundance of alien and native species are especially informative to assess impacts of alien species on biodiversity (see point iv).
(iii) **Trend**: The third aim relates to questions regarding a change—in either the abundance or the spatial distribution of a species—by ensuring some components of the temporal or spatial variation are captured in the data. Community science projects can be particularly useful for research addressing spread dynamics over large spatio-temporal scales (Preuss et al. 2014; Roy and Brown 2015), which would otherwise be unattainable. For instance, in Britain and Belgium researchers have been able to track the spread of the harlequin ladybird (*Harmonia axyridis*) with a large-scale community science survey (Adriaens et al. 2008; Brown et al. 2018). In Portugal and Italy, community science data revealed expansion rates of the Asian hornet (*Vespa velutina nigrithorax*) and the brown marmorated stink bug (*Halyomorpha halys*), respectively, facilitating the development of appropriate management strategies at the regional level (Carvalho et al. 2020; Maistrello et al. 2016). Given the predicted general increase in the number of alien species worldwide (Seebens et al. 2020) and the way projected climate change is expected to alter species distributions (Essl et al. 2019), community science data will certainly play a central role in informing future predictive models (Kress et al. 2018).
(iv) **Impact**: The fourth aim relates to identifying the impacts of alien species. In cases where impacts are investigated, they are often inferred from correlations with affected native species in terms of population trends, namely abundance, and distributional changes, or in some cases, other indirect measures such as numbers of dead trees or water quality (Colléony and Shwartz 2020; Diamond and Ross 2019; Guyot et al. 2015; Koenig et al. 2013; Roy et al. 2012). Such trends should be interpreted with caution as multiple causative agents of the decline of native species and populations may not be captured in the study (Byers 2002), potentially leading to an overestimation of alien species impact. However, information on spatio-temporally co-occurring species, including species from the same guilds, host or food plants, overlapping phenology etc., is useful to assess potential impact of an invader. For instance, Adriaens et al. (2008) calculated niche overlap indices which informed ecological risk assessment for an invasive alien ladybird (Kenis et al. 2017). Future focus on the interactions between native and alien species may be more informative for discerning impacts of alien species, particularly alongside mechanistic experimental studies.

### Identifying the different sources of uncertainty in community science projects

**Fig. 1:**
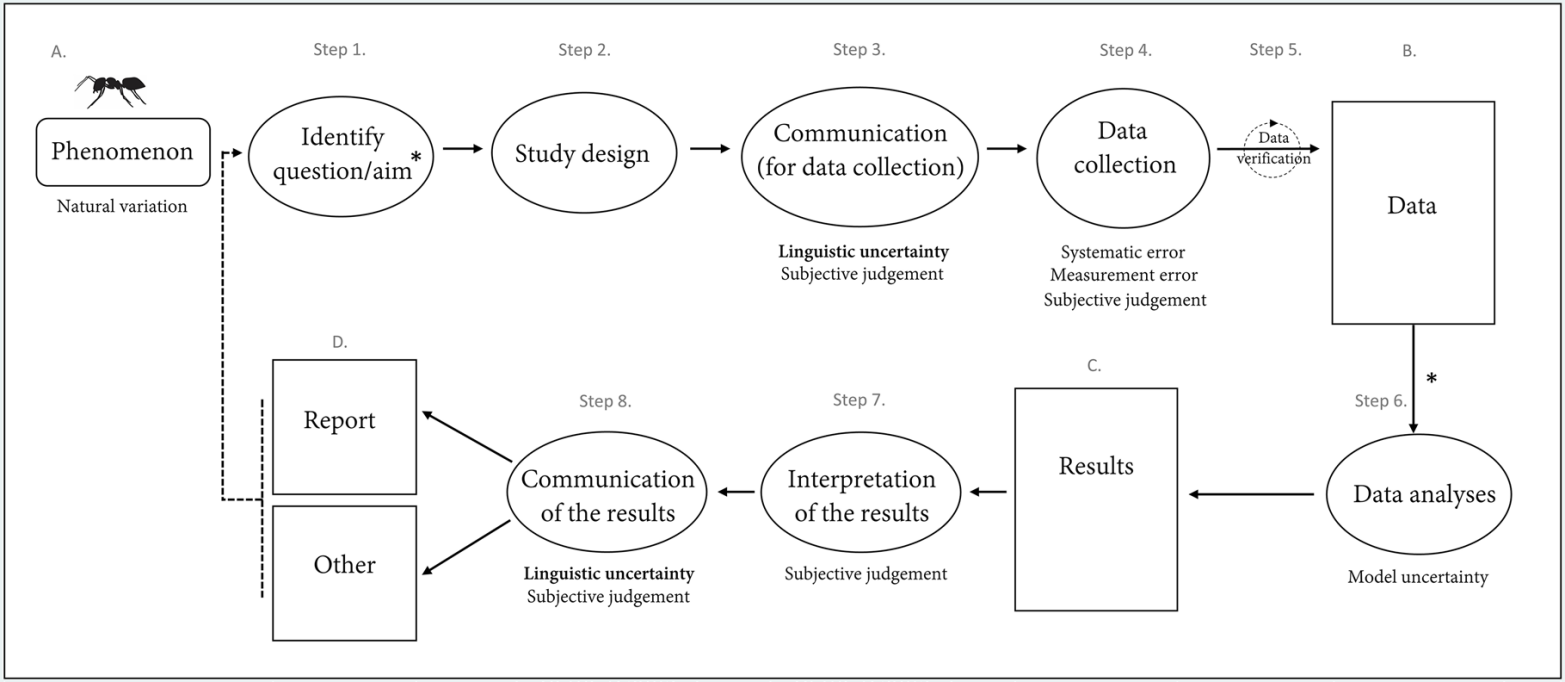
Schematic of a generalised scientific process identifying where differences sources of uncertainty emerge in context to community science projects related to alien species. The different steps, or actions, are outlined and encapsulated within ovals, whereas the text in rectangles indicate outcomes generated by the actions. The process begins at A., with the occurrence of some phenomenon (e.g., arrival, spread) of an alien species to be investigated. Sources of uncertainty (Regan et al. 2002) are indicated where they arise across the process: the specific types of epistemic uncertainty are listed and linguistic uncertainty is identified in general. During the communication for both data collection (Step 3) and the results (Step 8), subjective judgement is relevant as it may influence the message made by the communicator(s) and thus the way the recipient audience perceives the information. The asterisks at ‘Identify question/aim’ (Step 1.) and between the ‘Data’ (B.) and ‘Data analyses’ (Step 6.) stages, indicate that for some projects, research questions may be generated post-data collection (e.g., projects that data mine biological databases). The dashed lines represent the potential for information learnt during any stage of the process to be integrated into subsequent actions for longer-term projects allowing the process to become refinements and improvements to be incorporated

Before addressing the different sources of uncertainty, it is important to understand the process of scientific inquiry in order to identify where uncertainty can emerge and propagate to subsequent steps. We created a conceptual figure to demonstrate how community science projects can follow a generalised process of scientific inquiry, allowing potential sources of uncertainty to be identified at the various steps (Fig. 1). First, the occurrence of a phenomenon related to an alien species will initiate the motivation to ask a scientific question (Fig. 1; A). Identifying this question and developing a study to investigate the phenomenon (Fig. 1; Steps 1 and 2) may be done before data collection (Fig. 1; Step 4), either by professional scientists, community members or through co-creation. Alternatively, data may already exist, for example in online biodiversity databases, in which case considering the steps prior to the stage of data analyses become less relevant as fewer sources of uncertainty may be controlled. After data have been gathered, they may or may not be verified by experts (Fig. 1; Step 5), prior to analysis and interpretation (Fig. 1; Steps 6 and 7). This information is then ultimately communicated to stakeholders and sometimes the scientific community, for instance in the form of a report or publication (Fig. 1; Step 8; D). For projects spanning longer temporal scales, information learnt during the process may be integrated into subsequent actions allowing the refinement and improvement of the different stages via a feedback loop. It is important to note that uncertainties arising at each step can propagate to the following research steps, and thus become compounded at the subsequent stages.

Using the taxonomy of uncertainty outlined by Regan et al. (2002)—where uncertainty sources are classified as either *linguistic* or *epistemic* (Table 1)—we identify where different sources of uncertainty emerge during the process of scientific inquiry, in the context of citizen science for alien species research (Fig. 1).

**Table 1:** Epistemic and linguistic uncertainties (Regan et al. 2002) relevant to community science in context of research addressing alien species

**Epistemic uncertainties**	
*Natural variation* Relates to the changes (usually related to space and time) which are naturally and inherently present but often difficult to predict. Community science projects may be able to help reduce such biases, particularly related to natural spatial variation, by upscaling data collection. Example: Populations of alien species vary in different demographic attributes due to natural changes in fecundity and mortality.	
*Measurement error* Occurs due to imperfect measuring equipment and observation techniques, including when the individual and/or equipment causes the error. Example: A participant under- or over-estimates the number of individuals of a species on site. The Global Positioning Satellite (GPS) of a smartphone is slow and records an observation in a different kilometre square or with high associated coordinate uncertainty.	
*Systematic error* Arises due to biases in sampling procedure or measuring equipment. Example: For example, two species A and B look relatively similar. A volunteer recorder consistently records the presence of species A as the presence of species B as they do not realise that there are two different species; or, a volunteer recorder incorrectly sets up a GPS device and now all locations that the participant records are systematically wrong.	
*Model uncertainty* Results from the necessity to represent the ‘true’ situation through the use of simplified models. Model uncertainty is generated from the fact there are a multitude of drivers that affect a process, and we will never capture the true scenario. Model uncertainty may be reduced through model validation methods (Zurell et al. 2010) and ensuring that findings are interpreted within the limits of the model.	
*Subjective judgement* Arises through the interpretation of information. This is relevant from the perspective of both the project co-ordinators and the audience (e.g., volunteer recorders) receiving the information. Linguistic uncertainties may exacerbate subjective judgement. Example: A scientist believes that changes caused by an alien species in an ecosystem (e.g., soil pH) are generally deleterious for native species and therefore describes them in the report as detrimental for the ecosystem.	
**Linguistic uncertainties**	
*Vagueness* Language that permits borderline cases; common when using linguistic categories that underpin continuous measurements. Example: Asking volunteer recorders to provide linguistic size class categories, such as small, medium and large, for a specific species observation, may lead to inconsistencies.	
*Context dependence* When the context under which something is required to be completely understood is absent. Example: Species may be thought of as either native or alien, depending on their geographical range. For instance, species translocated within a country may be perceived as native by some yet alien by others. Understanding the native range is necessary for context to determine if it is alien.	
*Ambiguity* Where more than one meaning for a word or phrase may be interpreted and it is not clear which meaning is correct. Example: The term ‘invasive’ can be interpreted differently as current definitions use it to refer to alien species that are established and widespread across a landscape with no reference to impact, or alternatively, alien species that are perceived to have deleterious impacts.	
*Under specificity* When there is an unwanted generalism and information is not clear due to the lack of detail. Example: Failure to clearly explain to project participants the level of details necessary for each species observation may lead to incomplete records and data gaps.	
*Indeterminacy of theoretical terms* Occurs because language is imprecise, and words can change meaning with time. Example: Species names (both their scientific nomenclature and common names) can change over time, causing confusion to those who were aware of their previous names.	

#### Linguistic uncertainty

During any step of the project that requires communication, uncertainty can manifest through imprecise language (from the communicator(s)) or misunderstanding (audience), leading to confusion and misinterpretation of messages (Fig. 1; Steps 3 and 8). Linguistic uncertainties are not mutually exclusive; that is, words and phrases may comprise more than one of the different types of linguistic uncertainties (Table 1). For example, a phrase may be simultaneously vague and ambiguous, or ambiguous and contain uncertainty due to lack of specificity. It is important to note that linguistic uncertainty from communicators can amplify uncertainty due to subjective judgement (see section on epistemic uncertainties below) which arises due to the individual interpretation of information by the audience. Given that subjectivity refers to personal feelings and opinions rather than facts it may be fair to expect that inputs of uncertainty due to subjective judgement will be magnified through the participation of many individuals during data collection when compared to projects under the common scientific framework.

The first step where linguistic uncertainties will initially be introduced is during the ‘Communication for data collection’ stage (Fig. 1; Step 3), where project coordinators will specify information pertaining to the project, such as the rationale behind the research and the methods in which they require participants to collect data. Under the common scientific framework, communication is usually restricted to far fewer data collectors that would be recruited based on their level of expertise to collect data and are usually provided with in-person training. The increased expertise acquired through training and the lower number of people involved makes it easier to control for linguistic uncertainties. The subsequent step involving communication where linguistic uncertainty can arise is at the ‘Communication of the results’ step (Fig. 1; Step 8), when the findings of the project are communicated either in the form of a report or directly to an audience. The types of linguistic uncertainty relevant here differ from other scientific projects in the sense that they might require communication to a broader audience. For instance, findings from community science projects addressing alien species may be published in academic journals or communicated by other means to decision makers and stakeholders. Most importantly, results and project conclusions should be communicated to all participants in a way that can be clearly understood regardless of their individual level of expertise and scientific knowledge (see section four).

A key consideration is that scientific terminology may be unfamiliar and interpreted differently by community science participants due to the uncertainty associated with technical terms and phrases. Indeed, similar problems arise within the use of ordinary language where definitions can have varying meaning due to cultural differences. This is particularly applicable to the invasion science lexicon which is known for its value-laden terminology in some contexts (Verbrugge et al. 2016). For example, although the terms ‘alien’, ‘exotic’ and ‘non-native’ are frequently used interchangeably, research has demonstrated that ‘exotic’ is more often perceived more favourably and associated with beneficial impacts (Kapitza el al. 2019). Thus, carefully selecting the terminology used will be important to consider for projects that involve individuals from wider geographical scales and particularly if projects necessitate information being translated into additional languages.

#### Epistemic uncertainty

In any scientific project, epistemic uncertainty is always present as natural variation in the observed phenomenon (Fig. 1; A; Table 1). Because community science projects can facilitate the collection of data over greater spatial and temporal scales due to the increased ‘people power’, such uncertainties may be better accounted for compared to other less-intensive studies. Whilst experts are thought to consistently collect high quality data with reduced measurement error, numerous studies have demonstrated community members can have similar capabilities in terms of both accuracy and reliability (Crall et al. 2011; Kallimanis et al. 2017). However, this is highly dependent on the study system and research question at hand, given that some species will inherently be more difficult to detect and/or identify than others (Brandon et al. 2003; Forrester et al. 2015). Additionally, poorly communicated background information and instructions may lead to confusion and inconsistencies among volunteer recorders during the data collection stage.

In all studies, epistemic uncertainty arises during the data collection. Here, these uncertainties will most notably arise in the form of measurement error, systematic error, and subjective judgement (Fig. 1; Step 4; Table 1). Compared to studies conducted under the common scientific framework it might be expected that such uncertainties are amplified given the increased number of individuals contributing to data collection. In community science projects, there are more chances for individual observer-level error to be introduced during the data collection (e.g., misidentification of a species) and recording (e.g., incorrectly entering data into a spreadsheet). This introduces additional variation when compared to other methods linked to when, where and what volunteers record (Boakes et al. 2016). Furthermore, the introduction of subjective judgement may lead to taxonomic, geographic, and temporal biases. For instance, observer preferences for particular taxa—which can be influenced by culturally related preferences or individual interests (Ressurreição et al. 2012)—result in detection biases. In this sense, subjective judgement can create systematic error whereby individuals will intentionally include or exclude observations. Such biases have been demonstrated by Caley et al. (2020), who found volunteer recorders tended to preferentially log opportunistic insect occurrence reports for species with more striking physical features. Similarly, volunteer recorders may be more likely to visit some localities over others for various reasons, such as their proximity to home, ease of access or preference of habitat type (Petersen et al. 2021; Tye et al. 2017), leading to data with strong spatial biases (Geldmann et al. 2016). There may also be biases in the weather conditions or time of day and year when volunteer recorders collect the data (Baker et al. 2019); these spatial and temporal biases can lead to natural variation being poorly captured in the data, creating knowledge gaps (Regan et al. 2002). Additionally, Boakes et al. (2016) showed the recording behaviour itself can introduce bias which is considered separately from volunteer’s natural preferences for taxa and places. This is certainly relevant for the recording of high-profile invasive alien species which receive a lot of media coverage, are often well known to everyone and therefore have a higher recordability.

Subsequent forms of epistemic uncertainty arising after data collection include model uncertainty during the stage of data analyses (Fig. 1; Step 6) and additional subjective judgement arising when research findings are interpreted and communicated (Fig. 1; Steps 7&8). Model uncertainty is inherent to all scientific research given the necessity to describe biological phenomena using simplifications. Every time we collect data to make inferences to describe the true state, natural variation leads to model uncertainty (Regan et al. 2002). Once data have been analysed, subjective judgement is generated by the project coordinators when they interpret data and communicate findings and when the audience (e.g., volunteer recorders) interprets this information (Fig. 1; Step 8; D).

### Reducing uncertainty

All research will inevitably be associated with various forms of uncertainty; however, if these are appropriately considered—in terms of where and why they arise—different approaches may be taken to reduce the overall uncertainties that may be relevant when designing a project and during the downstream data analyses. We recognise that linguistic forms of uncertainty (Table 1) are of great importance given their ability to contribute to the emergence of subjective judgement among participants (see section above). Thus, our overall recommendation to reduce forms of linguistic uncertainty and subjective judgement in this context is to be mindful of the language during all stages where communication is involved. Specifically, avoiding the use of jargon-laden language where possible and in cases where this is unavoidable, providing simple, clear and concise definitions for scientific terms. Ideally, a reciprocal dialogue between participants and project coordinators should be established to allow volunteer recorders to ask questions and clarify aspects they may not initially understand. In doing so, the communication approach may be refined in the future as the feedback is integrated into the project (Fig. 1).

Below, we focus and discuss different ways to reduce epistemic uncertainty by: (i) increasing the quality of data generated by participants, (ii) choosing an appropriate experimental design to account for uncertainty in the analyses, while we provide (iii) specific examples to account for uncertainty.

#### Increasing quality of data generated by participants

The ability of community science participants to accurately collect data will depend largely on the question to be addressed. Some projects require participants to have more specific identification skills that may be improved through training or practice alone (Gallo and Waitt 2011; Kampen et al. 2015; Starr et al. 2014). One major issue is that, generally, novice volunteer recorders are more likely to misidentify or overlook species compared to professional scientists (Austen et al. 2016; Falk et al. 2019; Galloway et al. 2006), which can lead to uncertainty in single observations regarding species identification and presence at specific locations. The ability to accurately detect and identify species may vary significantly among volunteer recorders depending on the individual skills of participants; for instance, some individuals may be amateur experts with abilities equal to professional scientists. Importantly, however, it should be noted that some biases may become more, or less, prevalent as the skill-level increases among participants. For instance, Farmer et al. (2012) found a tendency for more false positives of rare species to be recorded by participants with higher expertise. In contrast, Groom and Whild (2017) found false positives to be uniformly distributed among observers of different expertise, yet both studies reported higher frequencies of false positive detections for rarer species when compared to more common species. Increasing participants’ observational skills, in the aim of reducing false negative and false positive detections, may be directly addressed by providing training and feedback (but see Feldman et al. 2018), although such an option is often not feasible for many community science projects. In some cases, the development of online tools to support learning may provide an accessible way to improve community science skills as well as to promote engagement and reach educational goals. Online tools that provide volunteer recorders a platform to interact (e.g., iNaturalist; www.inaturalist.org) may also help to increase individual competencies through peer feedback.

Currently, the majority of community science projects focusing on the recording of alien species have a verification step, whereby data collected by participants (most often images or specimens) are confirmed by experts (Schade et al. 2019; Wiggins et al. 2011; Supplementary material). For example, during a survey on 103 alien species community science projects in Europe, 89 projects indicated using validation procedures (Alien-CSI consortium, (Price-Jones et al. 2022)). Generally, the most prominent approaches for validation of community science data are expert- and peer-validation, sometimes aided by automatic filtering techniques (e.g., through data mining algorithms, artificial intelligence) which can address random variation, such as outlier detection (Balázs et al. 2021; Wiggins et al. 2011). Model-based quality assessment can tackle errors using an explicit model of variation in space and time. For example, Kelling et al. (2015a, b) indexed eBird (www.eBird.org) observers variability using species accumulation curves to account for observer skill and improve data quality post-hoc. The relatively labour-intensive step of data verification is often necessary to ensure data quality, but future identification will likely become more efficient through the use of machine learning based on imagery, acoustics, and environmental DNA at both the individual and landscape-level (Demertzis et al. 2018; Demertzis and Iliadis 2017; Kganyago et al. 2018; Milián‐García et al. 2021; Terry et al. 2020).

#### Choosing an experimental design that allows errors to be estimated and accounted for downstream

**Fig. 2:**
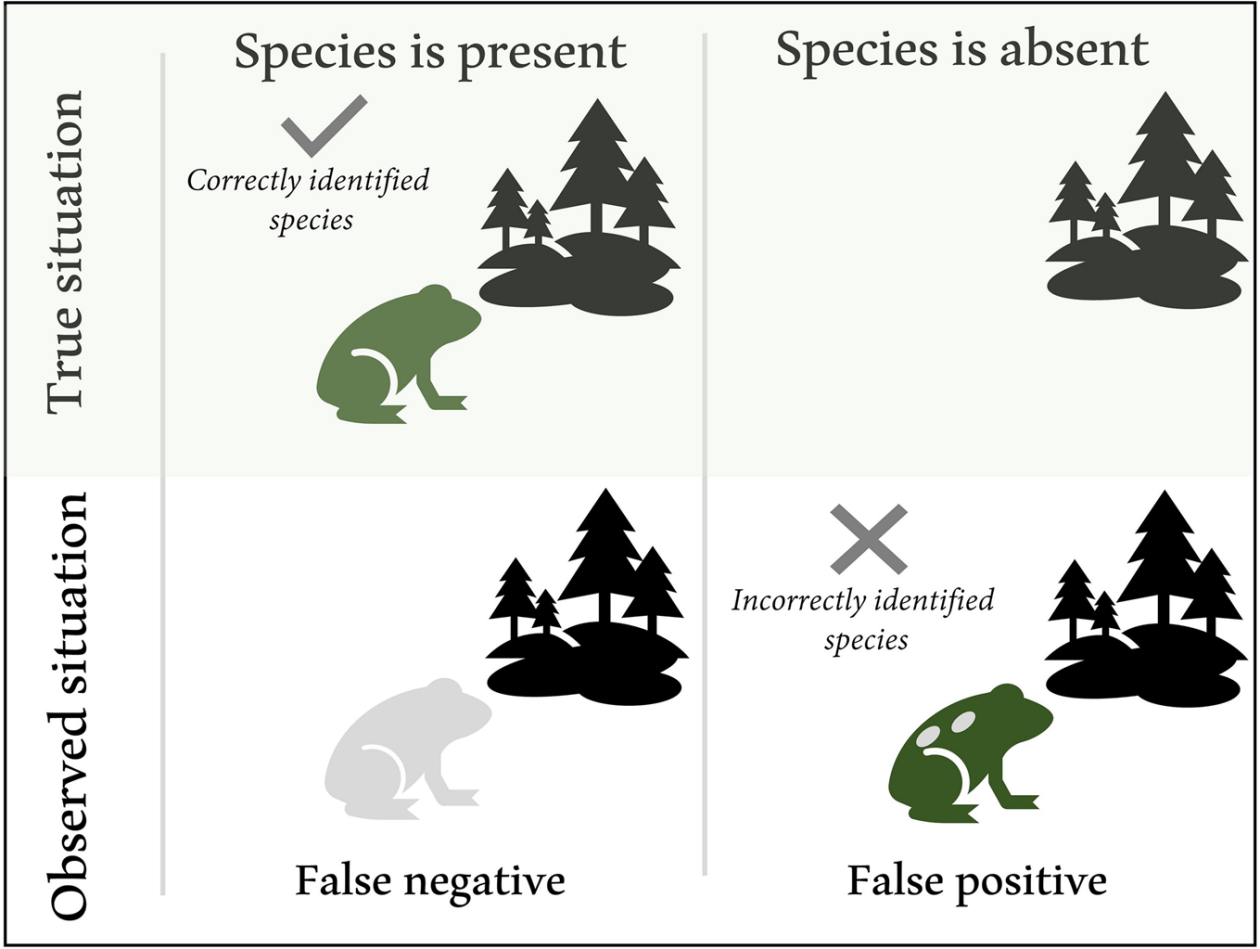
Two forms of detection error illustrated using an example of an alien frog at a specific location. False negatives (Type II error) occur when an observer does not detect the target species (the “green” frog) that was indeed present, because i) observers are looking in the wrong place (e.g., the species occurs on plant A, but the observer only looks on plant B), or the species is ii) cryptic or hidden, or, iii) incorrectly identified (in our example, the target species is misidentified as another species). False positives (Type I error) occur when an observer incorrectly detects the target species (usually based on a misidentification – here, the “spotted” frog is mistaken for the target species)

**Fig. 3:**
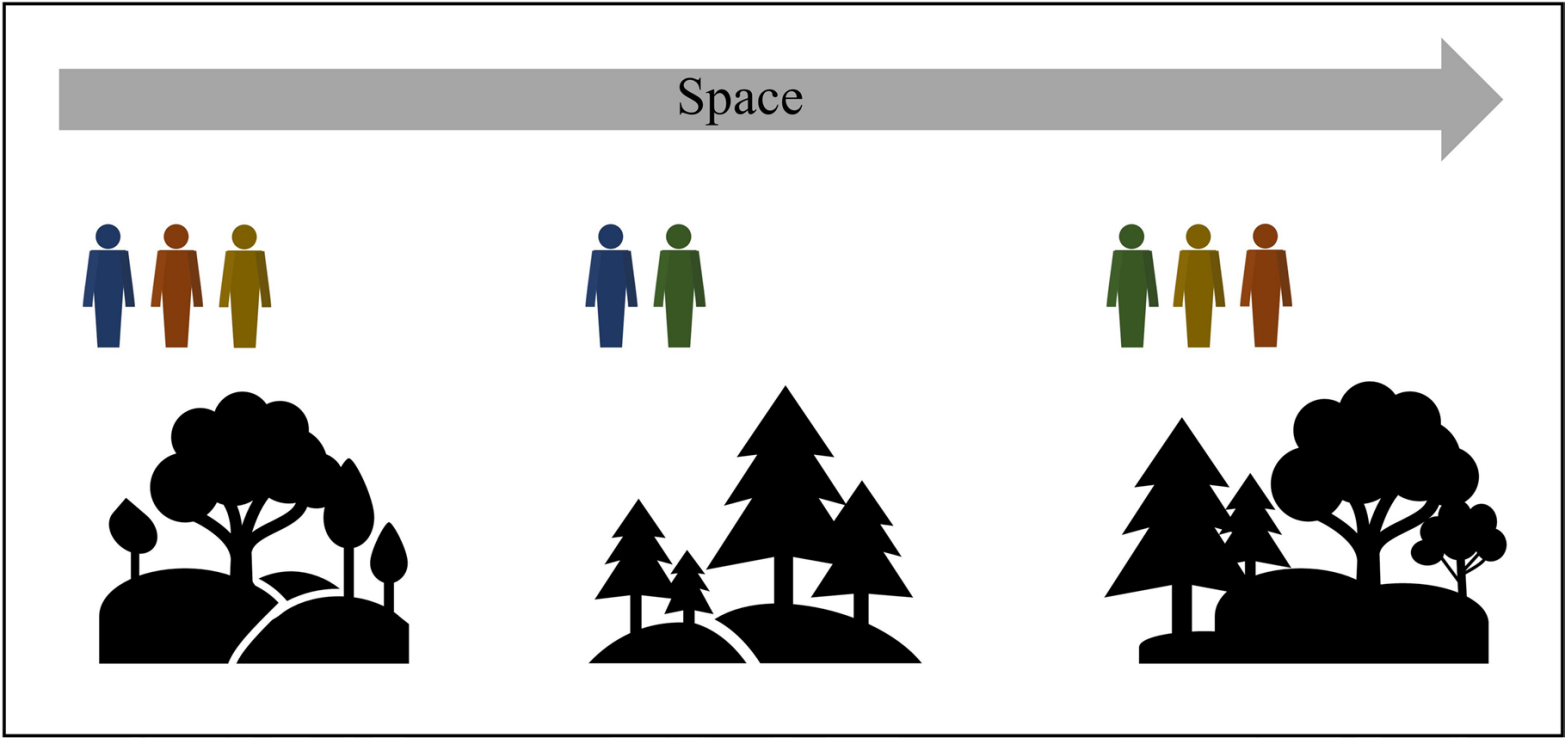
Detection biases may be accounted for by obtaining information about individual observer’s detection rates. Some observers may be more likely to detect a species. To learn error rates, studies should be designed such that different locations are visited by more than one observer (illustrated by figures of different colours). Not all projects will lend themselves to such a design; there may be few participants and/or participants may be unable to visit multiple locations

When considering the experimental design for a project where a community scientist will survey a specific location (or site) for the presence of an alien species, we can distinguish two types of observation errors (Fig. 2): the alien species is present but is not detected/identified (i.e., a false negative detection), or the alien species is not present but recorded due to misidentification or false reporting (i.e., a false positive detection). Although proper training can minimise these errors, they are unlikely to be eliminated and therefore need to be accounted for statistically, especially for species and life stages not easily identifiable. This is possible if the rates at which these errors occur are either known or can be learned from the data, with the possibility of the latter depending on the experimental design (Fig. 3).

Error rates of any kind may only be learned from replicate data points. Take, for example, a volunteer recorder visiting the same location multiple times. If the alien species is present at that location, the fraction of visits at which it was not detected provides information about the rate of false negatives. Similarly, if a volunteer recorder reports the alien species at several locations from which no other volunteer recorder has ever detected it, that individual must either be superior at detection or otherwise misidentifies the species frequently. A key realisation from this is that error rates can only be learned properly if absence data are collected: if a volunteer recorder only reports visits that resulted in an observation, the data contains no information about the probability of detection. In cases where absence data (i.e., non-detections) cannot be collected directly, they may be estimated, for instance through observer characteristics such as reports of common species, the number of visits to a location, the length of a species list or other such covariates (e.g., Bradter et al. 2018; Kelling et al. 2015a, b; Lele et al. 2012).

In the case of high error rates, uncertainty may be reduced by focusing on hierarchical parameters, i.e., model parameters that govern other parameters of the model (Box 1). The reason is that for hierarchical parameters, many data points are collectively informative, and this information can be exploited if error rates are either known or can be estimated accurately from the data. The fraction of locations at which an alien species is present, for instance, may be estimated accurately, even if the presence at individual locations is highly uncertain (Box 1). When designing community science projects, we thus recommend identifying the most relevant hierarchical parameters and to choose an experimental design most suitable for those.

**Box 1:** Inferring hierarchical parameters

**Fig. 4:**
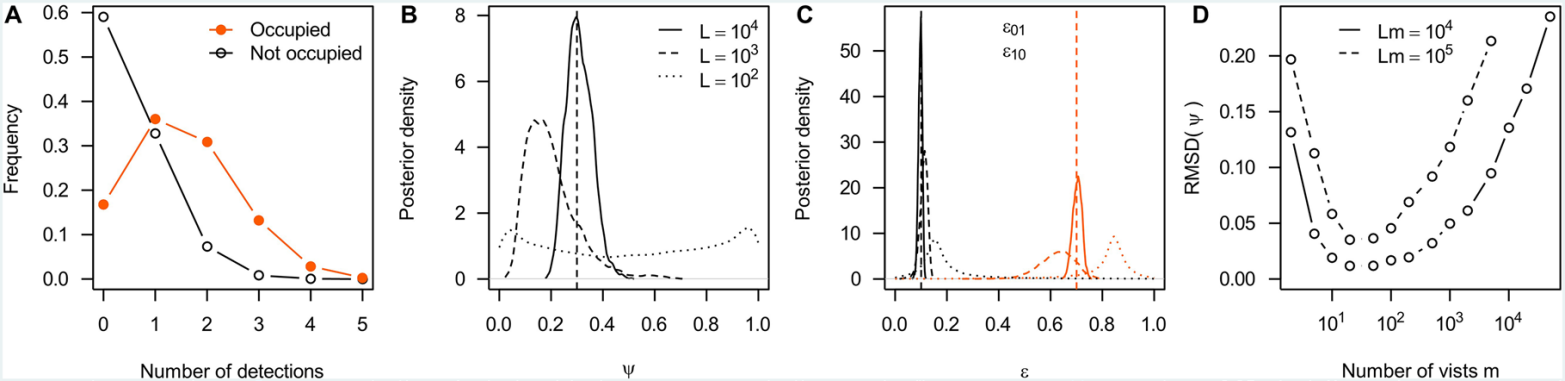
Illustration of the hierarchical model from Box 1 with parameters $\psi =0.3$, ${\epsilon }_{01}=0.1$ and ${\epsilon }_{10}=0.7$ (dashed vertical lines). **A**: expected distributions of the number of reported detections at occupied (filled, orange) and not occupied locations (open, black) for $m=5$ visits per location. **B** and **C**: Posterior distributions on $\psi$ (**B**), ${\epsilon }_{01}$ (**C**, black) and ${\epsilon }_{10}$ (**C**, orange) for data simulated at $L={10}^{4}$ (solid), $L={10}^{3}$ (dashed) and $L={10}^{2}$ (dotted) locations with $m=5$ visits each. **D**: Accuracy of inferring $\psi$ as quantified by the root mean squared deviation (RMSD) of the posterior means of $\psi$ across 100 replicate simulations for different combinations of locations L and visits $m$ for $Lm={10}^{5}$ (solid) and $Lm={10}^{4}$ (dashed) total number of visits

As an example, consider a project in which volunteer recorders report detections and non-detections of an alien species at a large number of locations. Rather than inferring whether a location is occupied (i.e., the alien species is present) for each location individually, such data may be modelled using hierarchical parameters that govern the distribution of occupied locations. For instance, one might introduce the hierarchical parameter $$\psi$$ that reflects the fraction of locations that are occupied. To illustrate this, consider a project in which volunteer recorders visit $$L$$ locations $$m$$ times each. Let $${d}_{i}$$ reflect the number of visits at location $$l=1,\dots ,L$$ that resulted in a detection, and the remaining $$m-{d}_{l}$$ in a non-detection. Let us further denote by $${z}_{l}$$ wheter location $$l$$ is occupied ($${z}_{l}=1)$$ or not ($${z}_{l}=0)$$ and by $${\epsilon }_{10}$$ and $${\epsilon }_{01}$$ the false negative and false positive detection rates, respectively. Under this model, $$P\left({d}_{l}|{z}_{l},{\epsilon }_{01},{\epsilon }_{10}\right)=\left\{\begin{array}{c}\left(\genfrac{}{}{0pt}{}{m}{{d}_{l}}\right){\epsilon }_{10}^{{d}_{l}}{\left(1-{\epsilon }_{10}\right)}^{m-{d}_{l}} \quad \text{if} \quad {z}_{l}=0,\\ \left(\genfrac{}{}{0pt}{}{m}{{d}_{l}}\right){\left(1-{\epsilon }_{01}\right)}^{{d}_{l}}{\epsilon }_{01}^{m-{d}_{l}} \quad \text{if} \quad {z}_{l}=1.\end{array}\right.$$ As an example, we consider the case with $$m=5$$ visits per location, $${\epsilon }_{01}=0.1$$ and $${\epsilon }_{10}=0.7$$. As shown in Fig. 4A, accurately identifying occupied locations is difficult under these parameters: the most likely data at occupied locations is $${d}_{l}=1$$, which is almost equally likely to get at non-occupied locations as well. To infer the hierarchical parameters $$\psi$$, $${\epsilon }_{01}$$ and $${\epsilon }_{10}$$, we integrate out $${z}_{l}$$ to obtain the relevant likelihood $$P\left(d|\psi ,{\epsilon }_{01},{\epsilon }_{10}\right)=\prod_{l=1}^{L}\left[P\left({d}_{l}|{z}_{l}=0,{\epsilon }_{01}, {\epsilon }_{10}\right)\left(1-\psi \right)+P\left({d}_{l}|{z}_{l},=1{\epsilon }_{01}, {\epsilon }_{10}\right)\psi \right].$$ In Fig. 4B,C, we show Bayesian estimates of the parameters $$\psi$$, $${\epsilon }_{01}$$ and $${\epsilon }_{10}$$ from data simulated at $$L=100$$, $$L=\mathrm{1,000}$$ or $$L=\mathrm{10,000}$$ locations, confirming that these hierarchical parameters can be inferred rather accurately if sufficient locations were surveyed. Importantly, however, error rates can only be accurately learned if there are enough sites with multiple detections and hence sufficiently many visits. For a fixed number of visits, estimation errors are therefore minimized for intermediate number of visits per location, for the error rates chosen here at about $$m=20$$ (Fig. 4D).

#### Accounting for uncertainty: specific examples

In the following section, we discuss approaches to learn and account for detection errors when assessing the distribution, abundance, and trends of alien species, related to each of the key project aims identified above.

**Distribution:** We distinguish two experimental designs to delineate the distribution of alien species. In the first design, volunteer recorders are asked to report potential sightings of the alien species without being instructed where to look. In the second design, volunteer recorders are asked to survey specific locations and to report whether or not the alien species was detected (e.g., Epps et al. 2014). These designs differ fundamentally in the error rates that may be learned. Since non-detections are not reported in the first design, no information on search effort is available, meaning we cannot infer error rates from these data alone. This is a general problem of presence-only data, and existing methods to infer species distributions from such data assume that presences and absences are predicted well by ecological covariates (e.g., Guisan et al. 2017; Renner et al. 2015). For rare alien species at the beginning of their invasion, however, environmental covariates may not be good predictors: their relatively recent introduction means they have likely only covered a small, and not necessarily representative, part of their environmental niche space. Inferring the spatial distribution of an alien species under such an experimental design thus requires the verification of reports and evidence by experts unless search effort can be estimated from other covariates such as reports of more common species, the number of visits, or the species list length (Isaac et al. 2014; Szabo et al. 2010).

By contrast, when non-detections are reported along with detections, error rates and species distributions can be estimated jointly. Occupancy models (MacKenzie et al. 2002) are the most frequently applied method to achieve this using community science data (reviewed extensively in Altwegg and Nichols 2019; Dennis et al. 2017; van Strien et al. 2013). The measure of interest under these models is the distribution of presences (occupancy) or absences of a species at surveyed locations, which are learned while accounting for false negatives by explicitly modelling and learning detection probabilities. Under the assumption of no false positive detections, these detection probabilities are readily learned if locations were surveyed multiple times: if the alien species was detected at a location at least once, all surveys at that location that did not result in detections must be false negatives (MacKenzie et al. 2002).

Occupancy models may also account for variation in detection rates among observers, but only if observers conduct surveys at different locations: if an observer surveys only a single location but never detects the target alien species, it may be because the alien species is not present at this location, or because the probability of the observer detecting the species is low (i.e., a high false negative error rate). The latter would be concluded if the same observer reported non-detections at locations where others did spot the alien species. If such a design is not feasible, variation in detection rates may still be accounted for by modelling them through covariates correlated with an observer’s level of training, the search effort spent at a location, or both (Johnston et al. 2018; Altwegg and Nichols 2019).

If the number of surveys per location is too low to accurately infer local presences or absences, hierarchical parameters may be learned. These typically include the fraction of locations at which an alien species is present, and ecological covariates predicting local presences and absences (Johnston et al. 2018; Altwegg and Nichols 2019). Compared with other species, however, the latter may be less useful for recently introduced alien species as their distribution may be less determined by characteristics of the environment but more by their introduction history and patterns of dispersal.

Classic occupancy models generally assume no false positives or that false positive rates are known. The reason is that false positives cannot be distinguished from true positives from reported detections alone. However, false positives are common in community science data, particularly for studies that aim at detecting recently introduced and hence rare alien species that are therefore easily misidentified (Groom and Whild 2017). To learn false positive rates in an occupancy setting, additional information must be available, either in the form of ground-truth at a subset of locations, or confirmed detections (e.g., by requesting to upload pictures of the observed individual(s) (Chambert et al. 2015; Vantieghem et al. 2017). The latter approach may be particularly appealing for community science data of recent invasions in which false positive rates are likely high, but a fair number of reported detections can be confirmed by experts.

**Abundance:** Inferring abundance is more challenging than occupancy: in the absence of false positives, a single detection is sufficient to identify a location as occupied, but a single detection may indicate a low abundance, a low detection probability, or both. If detection rates are low, however, variation in the frequency of detections at a location does provide information about variation in abundances between locations. The Royle-Nichols model (Royle and Nichols 2003), for instance, captures this information by assuming detection rates to scale exponentially with abundances. These models require the same experimental design as classic occupancy models.

**Fig. 5:**
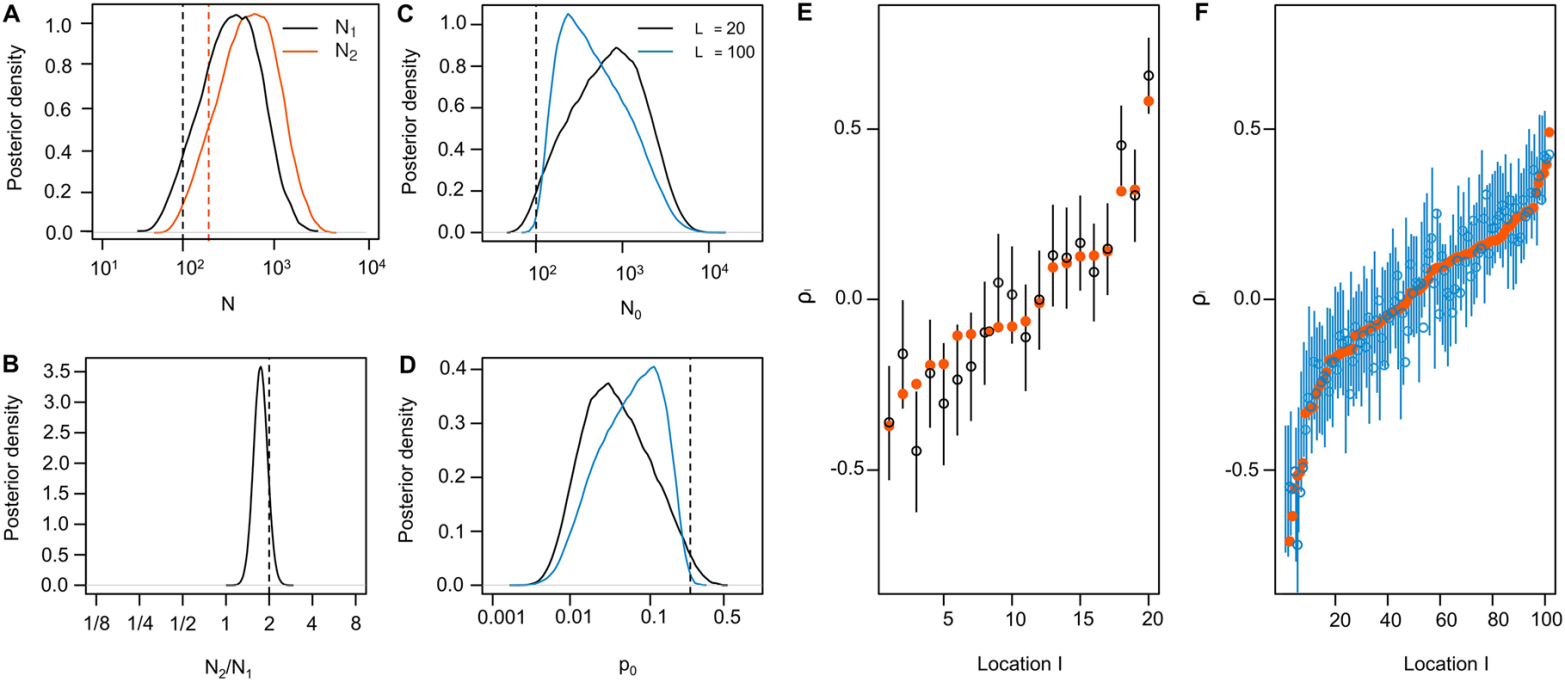
Inferring relative abundances under the models presented in Box 3. **A**–**B**: Posterior estimates of abundances from data simulated for five visits per location with ${N}_{1}=100$ and ${N}_{2}=200$ and detection probability $p=0.2$ (dashed vertical lines). **B**: Posterior distribution of the relative abundance of ${N}_{2}/{N}_{1}$ from the data of A. **C**–**F**: Posterior distributions on ${N}_{0}$ (**C**), ${p}_{0}$ (**D**) and the relative abundances ${\rho }_{l}$ (**E** and **F**, mean and 90% quantile, true values as orange dots) for each location $l$ under the multi-location relative abundance model outlined in Box 3 and from data simulated with ${N}_{0}=100$, ${\sigma }_{\rho }^{2}=0.2$, ${p}_{0}=-1$ and ${\sigma }_{\pi }^{2}=0.5$ and either $L=J=20$ (black, **E**) or $L=J=100$ (blue, **F**)

If an abundance survey is targeted to locations at which the alien species is expected to be common (i.e., there are numerous individuals), most visits might result in detections. In these cases, simple presence-absence data are not sufficient to distinguish locations. Rather, observers should provide an estimate of abundance. These estimates may be from direct observations such as the number of individuals or a measure of vegetation cover, biomass or density, or from indirect observations such as the number of nests, the presence or frequency of faeces or tracks, or a browsing index. The aforementioned count data, however, do not lend themselves easily to infer error rates as the parameters regarding abundances and detection probabilities are confounded: a low abundance location surveyed with a high detection probability may result in the exact same number of observations as a high abundance location surveyed with a low detection probability. As a result, joint estimates of abundances and detection probabilities are associated with large uncertainty, even from a large number of replicates (DasGupta and Rubin 2005, Box 2, Fig. 5A). As shown in Box 2, it may therefore be advisable to infer relative abundances only, as these can be learned more accurately and jointly with relative detection probabilities if observers visit multiple locations or if relative detection probabilities are well characterised by covariates, as was recently shown for camera trapping data (Ait Kaci Azzou et al. 2021).

**Box 2:** Estimating relative abundances

Consider a survey designed to quantify the abundances ${N}_{l}$ at locations $l=1,\dots ,L$ from abundances reported by observers $j=1,\dots ,J$ from a total of $V$ visits. Let ${d}_{v}$ denote the reported abundance during visit $v=1,\dots ,V$ conducted at location ${l}_{v}$ by observer ${o}_{v}$. Here, ${d}_{v}$ is affected by both the abundance ${N}_{{l}_{v}}$ at location ${l}_{v}$ as well as by the detection probability ${p}_{{o}_{v}}$ of observer ${o}_{v}$ such that $P\left({d}_{v}|{N}_{{l}_{v}},{p}_{{o}_{v}}\right)=\left(\genfrac{}{}{0pt}{}{{N}_{{l}_{v}}}{{d}_{v}}\right){{p}_{{o}_{v}}}^{{d}_{v}}{(1-{p}_{{o}_{v}})}^{{N}_{{l}_{v}}-{d}_{v}}$ is given by binomial sampling. Since ${N}_{{l}_{v}}$ and ${p}_{{o}_{v}}$ are confounded, estimating them individually is difficult (DasGupta & Rubin 2005). To illustrate this, consider a case with two locations with ${N}_{1}=100$ and ${N}_{2}=200$ surveyed $m=5$ times each by a single observer with detection probability $p=0.2$. As shown in Fig. 5A, the uncertainty associated with abundance estimated from that data under mild priors ${N}_{1},{N}_{2}\sim {\text{Exp}}(0.001)$ spans about two orders of magnitude. This is because the data is well explained by pretty much any abundance if paired with a corresponding detection probability and more informative priors would be required to constrain the range of possible values. However, there is considerable evidence that ${N}_{2}$ is about twice ${N}_{1}$ (Fig. 5B), illustrating that relative abundances may be learned accurately from such surveys. To benefit from this in a realistic setting, we here generalize the inference of relative abundances to many locations. Let us assume that the abundances ${N}_{l}={N}_{0}{e}^{{\rho }_{l}}$ are scaled by location-specific factors ${\rho }_{l}\sim N(0, {\sigma }_{\rho }^{2})$ that are themselves normally distributed with mean zero and variance ${\sigma }_{\rho }^{2}$. Similarly, we assume that the detection probabilities ${p}_{j}={\text{logistic}}({\pi }_{0}+{\pi }_{j})$ are scaled by observer-specific effects ${\pi }_{j}\sim N\left(0, {\sigma }_{\pi }^{2}\right)$ that are also normally distributed with mean zero and variance ${\sigma }_{\pi }^{2}$. Here, the logistic transformation ensures $0\le {p}_{j}\le 1$. We further enforce the conditions $\frac{1}{L}{\sum }_{i}{\rho }_{l}=0$ and $\frac{1}{J}{\sum }_{j}{\pi }_{j}=0$ by scaling ${N}_{0}$ and ${p}_{0}$ accordingly. If observers do not visit multiple locations, the ${\pi }_{j}$ need to be modelled using informative covariates. We conducted simulations with ${N}_{0}=100$, ${\sigma }_{\rho }^{2}=0.2$, ${\pi }_{0}=-1$ and ${\sigma }_{\pi }^{2}=0.5$, corresponding to an average detection probability ${p}_{0}={\text{logistic}}\left({\pi }_{0}\right)=0.27$. As shown in Figs. 5C and 5D, neither ${N}_{0}$ nor ${p}_{0}$ can be inferred accurately, regardless of whether $L=20$ or $L=100$ locations were surveyed by $J=20$ or $J=100$ observers visiting $m=5$ different locations each, corresponding to $V=100$ and $V=500$ visits, respectively. In contrast, the relative abundances are estimated well, and easily distinguish locations with high from those with low abundances (Figs. 5E and 5F).

In some cases, removal models may be appropriate for estimating population sizes in tandem with management efforts and community science participants. Modelling the removal (i.e., the depletion) of individuals of a population within a given area can enable the estimation of an unknown population size (Zippin 1958). This method requires coordinated removal efforts and thus would be highly suitable in cases where alien species population control is being performed in tandem with skilled community science participants (e.g., hunters); for instance, Davis et al. (2016) used removal models to estimate population density of the invasive pig (*Sus scrofa*) using aerial gunning data.

**Trend**: Of interest may be both trends in the distribution and trends in the abundance of an alien species. Common to both is that changes in the effective search effort between surveys must be accounted for: If a community science project is, for instance, successful in acquiring new participants, or if the participants gained additional experience in detecting the target species, an increase in the number of reported detections may not necessarily reflect an increase in the abundance of that species. A statistical approach to infer population trends must thus account for temporal variation in the effective search effort, either by modelling it explicitly or through informative covariates such as the number of active volunteer recorders or their rate in reporting more common species whose abundance is assumed not to change through time.

For repeated survey data resulting in reported detections and non-detections, occupancy models can be extended to trends in distributions explicitly with two additional parameters: the rate at which an alien species colonised previously non-occupied locations, and the rate at which it goes extinct at previously occupied locations (MacKenzie et al. 2003). Similarly, Royle-Nichols models can be extended to detect trends in species abundances by explicitly modelling population growth (Dail and Madsen 2011; Hostetler and Chandler 2015). These so-called multi-season models generally require similar experimental designs as their single-season analogues, but they differ in one key aspect: a design in which observers survey a single location is permissible, even if their level of training is not well reflected by covariates. The reason is that while observers vary in their detection probabilities, information about a change in occupancy state is contained also in the data of a single observer visiting the same location repeatedly, allowing for error rates to be integrated out (Link and Sauer 1997).

This is also true for surveys in which volunteer recorders report direct or indirect estimates of abundances: regardless of the detection probability of an observer, a change in abundance translates into a change in the expected reported abundances (with the exception of a detection probability of 0). Link and Sauer (1997) introduced such trend models for direct or indirect observations well characterised by Poisson processes (including the negative-binomial distribution for overdispersed data), for which Aebischer et al. (2020) recently introduced a Bayesian solution. Most count data are well characterised by Poisson processes, including surveys in which volunteer recorders report all detections without surveying specific locations. While such a design does not allow for easy estimation of error rates (see above), it may still result in an accurate inference of population trends, as we show in Box 3. A common drawback of existing methods to infer trends in abundance is their assumption of no false positives. While protocols involving expert or community-based validation procedures may reduce false positives to a minimum (Schade et al. 2019; Wiggins et al. 2011), we identify the development of methods that explicitly account for false positives as an important area of future research.

**Box 3:** Inferring trends in abundances

**Fig. 6:**
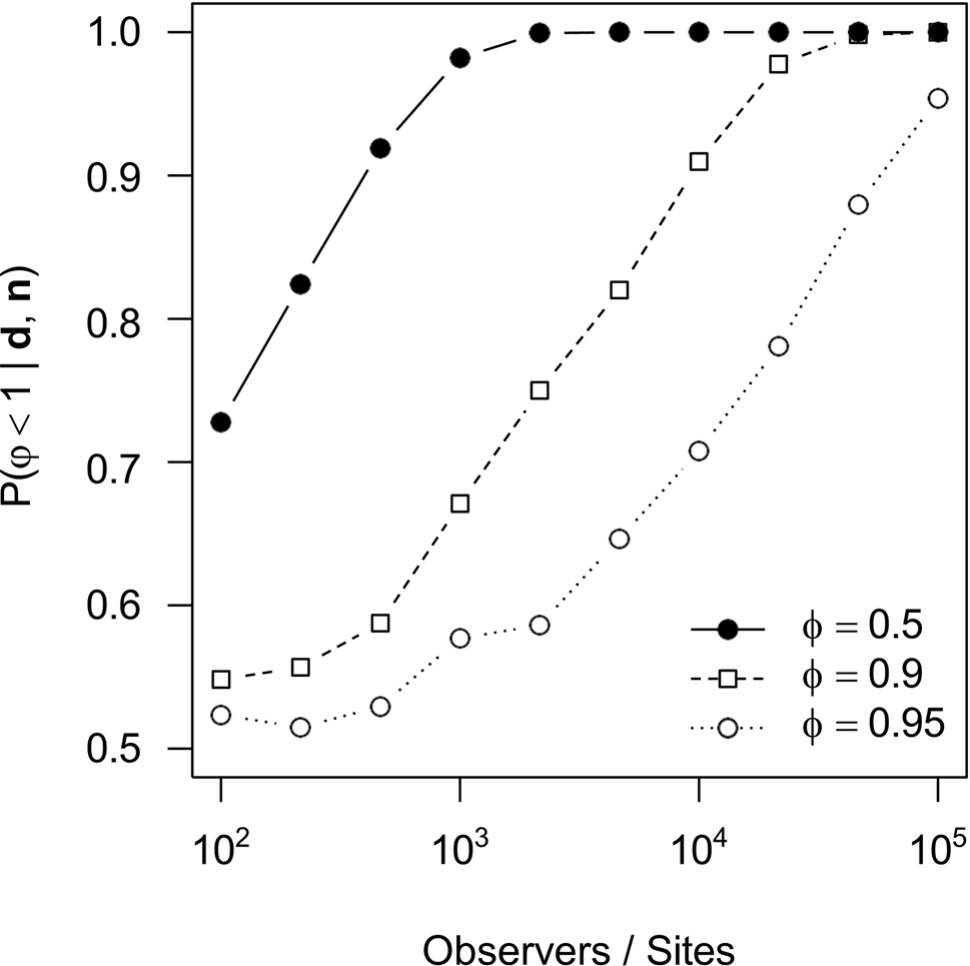
Power to identify trends in abundances. Shown are the mean posterior probabilities $P(\phi <1|{\varvec{d}},{\varvec{n}})$ given data ${\varvec{d}}$ conditioned on the total counts ${\varvec{n}}$ and reflecting the certainty that abundances declined across 1000 replicate simulations for different trends $\phi =0.5$, $0.9$ or $0.95$ as a function of the number of observers that each surveyed a single location with observer-specific detection probabilities and location-specific abundances as described in Box 4

We consider a design in which volunteer recorders are reporting GPS locations of all detections. We further assume that some information proportional to the spent search effort exists, such as the time volunteer recorders spend looking for the alien species or the number of reports of a commonly detected species. Let us denote by ${d}_{lj}(t)$ the number of detections reported by observer $j=1,\dots ,J$ during survey $t$ in a specific area $l=1,\dots ,L$, for instance a specific cell of a geographic grid, and let ${s}_{lj}(t)$ be a measure proportional to the search effort spent by observer $j$ in that area. Under such a design, ${n}_{lj}$ is likely well characterized by a Poisson distribution ${n}_{lj}\sim {\text{Pois}}({\lambda }_{lj}(t){s}_{lj})$ with unknown rates ${\lambda }_{lj}(t)$. Note that these rates are affected by the abundance at location $i$ as well as the detection probability of observer $j$ at that location, itself a potentially complex function of the training of the observer as well as characteristics of the location (e.g., vegetation). Assuming that the detection probabilities are constant across surveys (${\lambda }_{lj}\left({t}_{1}\right)={\lambda }_{lj}({t}_{2})$), a change in the rates is reflective of a change in abundances. The interest therefore lies in inferring changes in the rates, which are independent of location or observer-specific characteristics. For a case of two surveys at ${t}_{1},{t}_{2}$, we thus have ${\lambda }_{lj}({t}_{2})=\phi {\lambda }_{lj}({t}_{1})$ and wish to infer $\phi$ from the data of all observers and all locations. Following Aebischer et al. (2020), conditioning on the number of observations ${n}_{lj}={d}_{lj}\left(1\right)+{d}_{lj}\left(2\right)$ leads to the likelihood $P\left({\varvec{d}}|\phi ,{\varvec{n}}\right)\propto \prod_{l=1}^{L}\prod_{j=1}^{J}{{p}_{lj}\left(\phi \right)}^{{d}_{lj}\left({t}_{1}\right)}{(1-{p}_{lj}(\phi ))}^{{d}_{lj}({t}_{2})}$ with ${\varvec{d}}=({d}_{11},\dots ,{d}_{1J},\dots {d}_{LJ})$, ${\varvec{n}}=({n}_{11},\dots , {n}_{1J},\dots ,{n}_{LJ})$ and ${p}_{lj}\left(\phi \right)=\frac{{\lambda }_{lj}{\left(t1\right)s}_{lj}(t1)}{{\lambda }_{lj}{\left(t1\right)s}_{lj}\left(t1\right)+{\phi \lambda }_{lj}{\left(t1\right)s}_{lj}(t2)}={\left(1+\frac{{s}_{lj}(t2)}{{s}_{lj}(t1)}\phi \right)}^{-1}$ Importantly, this formulation gets rid of the nuisance parameters ${\lambda }_{ij}(t)$. Note further that no absolute estimates of search efforts are required: since only their ratio is relevant, any quantity proportional to the search effort will do. The posterior distribution of $\phi$ is readily inferred under Jeffrey’s prior (Aebischer et al. 2020). To illustrate this approach, we simulated data for observers that each surveyed a unique location during two consecutive surveys. As each location was surveyed by a single observer, detection probabilities and the abundances cannot be inferred individually without strong assumptions about their distribution (there are less data points than unknowns). However, a trend in abundance may still be identified. To show that, we simulated observers $j=1, \dots , J$ with detection probabilities ${p}_{j}\sim {\text{Beta}}(0.01, 10)$, their search efforts as ${s}_{j}\left(t\right)\sim {\text{Exp}}(0.1)$ and the abundances at their location as ${N}_{j}\left({t}_{1}\right)\sim {\text{Pois}}(10)$. Data simulated this way resulted in reported abundances ${d}_{j}({t}_{1})=0$ in $>99\%$ of all surveys, representative for community science projects targeting rare alien species. We then identified the power to detect a decreasing trend with $\phi =0.5$, $0.9$ or $0.95$ for different numbers of observers (and corresponding locations). As shown in Fig. 6, trends are reliably identified if sufficient observers participate, with stronger declines generally easier to identify. Obviously, higher detection probabilities, higher abundances or larger search efforts would all result in higher reported abundances and render trend identification easier. In case the assumption of constant detection rates it not possible, covariates accounting for variation can be folded into ${s}_{lj}(t)$ (Link & Saur 1997).

### Communicating uncertainty to participants and other stakeholders

Effective communication should be considered a central component of all community science projects (Garbarino and Mason 2016). It is necessary to achieve project objectives which will be different for the various actors involved (e.g., volunteer recorders, professional scientists, managers, policymakers and other stakeholders). In the context of alien species research, clear communication is not only important in the recruitment, engagement, motivation and retention of participants (Dickinson et al. 2012), but can be instrumental for the success of any management decisions (Falk et al. 2016). Highlighting the extent to which the data collected are used may be relevant to strengthen the engagement of community members in such projects. In a complementary way, there is an interest in communicating towards stakeholders and decision-makers in particular, to make them understand how much added value there is in taking into account data from community science as an element of evidence. These two aspects can act in synergy and reinforce each other (Groom et al. 2019). Effective communication during the early stages (i.e., the recruitment of participants and data collection before it becomes routine) will require an explanation of the aims and importance of the project. Participants should be made aware about what their contribution may lead to (e.g., eradication or management of a species, research to underpin management decisions, research on invasion dynamics or impacts of alien species) given the potential for individual participants to oppose management methods and outcomes. Instructions for participants should be clearly explained to reduce the potential for miscommunication and improve data quality throughout the project. Importantly, by understanding the way in which messages can become misconstrued, we can minimise additional uncertainties that may emerge during communication.

Central to effective communication is establishing a reciprocal dialogue between project coordinators and participants, which should ideally be based on a two-way process (Shackleton et al. 2019). Sustained engagement throughout the project, where participants are able to provide feedback and ask questions, enables project coordinators to refine their approach to identify issues, which need to be tackled to reach the intended educational, engagement and research outcomes (Druschke and Seltzer 2012). In recent years a number of best practice guidelines for communication have become available to project initiators (e.g., Veeckman et al. 2019).

Objective and efficient dissemination of research findings and the associated uncertainty (in a way that reduces the potential for the audience to misinterpret and potentially misuse information) should be a central aim of community science initiatives. Communicating with participants by providing feedback and presenting the research findings and their implications is an important obligation of project coordinators working within the community science framework (Vries et al. 2019). As project results, particularly regarding alien species, may be relevant to policymakers and managers (Groom et al. 2019; Lioy et al. 2019), the approach used to communicate findings and their uncertainty may require adaptation based on the intended audience (e.g., project participants, scientists, the general public or decision-makers).

Following a framework of uncertainty communication outlined in van der Bles et al. (2019), we identify the different components to consider when developing research and communication strategies in community science projects.

#### Who is communicating to whom?

A key element to acknowledge is how the relationship between communicator(s) and the audience can influence how uncertainty is perceived. The audience may come from culturally diverse backgrounds, hold different values and motivating factors, and have a varying degree of numerical and scientific literacy skills (Ganzevoort et al. 2017; Wright et al. 2015). Thus, although there may not be a one-size-fits-all approach to the form in which uncertainty is communicated, consideration should be made as to who is communicating to whom and how may this affect intended project outcomes. For instance, the relationship between the communicator(s) and the audience can be important from the perspective of whether the information being received and the person/organisation conveying it are considered trustworthy (van der Bles et al. 2019). Trust of the audience in the communicator is of utmost importance as the lack of trust can lead to a defensive stance or rejection even if the messages are true (Tuler and Kasperson 2013). This highlights the importance of selecting communicators based on their reputation with the audience and their ability to effectively engage with participants. Some projects aimed at large-scale participation may warrant the use of professional science communicators, and/or public figures of endorsement, to be involved or consulted during the out-reach phases of projects to build rapport, encourage participation and continued involvement in the project. More targeted projects may benefit from involving individuals with greater relatability with participants during communication stages. For example, if an alien species has a much greater probability of establishing within agricultural landscapes, involving one or more local farmers to act alongside project coordinators in a communication role may facilitate improved project outcomes. This could be particularly important if implications of the research may lead to management actions that require landowner support. Another option is to use project ambassadors i.e., participants of community science initiatives acting as role models for other participants and helping in promotion and community building (Druschke and Seltzer 2012).

#### What is being communicated?

Identifying exactly what we are uncertain about is necessary to then determine the way in which it should be communicated (van der Bles et al. 2019). Here, it should be noted that both the information that we are uncertain about and how it is expressed can influence the effect of the communication to the audience. When the data have the potential to be used for a specific purpose in decision making processes, it is important to have a good understanding of the information needs, how and to what extent the consideration of uncertainty may influence the decision making and steer the decisions. This helps to identify what needs to be communicated.

For community science projects that address aspects of alien species, uncertainty will stem from whether the collected data adequately capture the information required to answer the specific research question(s). Effectively, identifying the types of uncertainty that need to be communicated can help to determine how best to do so. For instance, does the uncertainty arise because of sampling variation across space or time (i.e., the experimental design), or rather because there is a lack of knowledge around the biology and dynamics of a given species (i.e., there is a general knowledge gap)? Further, how large are these uncertainties and how does that affect our confidence in the results? Different analytical techniques can be applied to derive measures of certainty around the data that are usually expressed through probability distributions or qualitative statements and may be communicated through various forms of graphical visualisations such as error bars and confidence intervals (Padilla et al. 2021). Identifying exactly what the source of uncertainty is will help to guide appropriate ways to communicate it and can affect how information is perceived (see below). Communicating these identified uncertainties to participants/stakeholders in a clear and transparent manner is critical to create and maintain trust in the results and the people who participated in the project. Importantly, potential conflicts of interest should be explicitly stated as participants may be sceptical of findings if they perceive a biased agenda.

#### To what effect?

The effect to which uncertainty is communicated will vary among the audience. This is due to the strongly subjective nature of interpreting information, based on not only what the message is but also in the medium or format in which it is conveyed (van der Bles et al. 2019). For instance, the various ways that uncertainty is visualised (e.g., error bars around a mean, boxplots etc.) are not consistently understood among people (Padilla et al. 2021). This can be shaped by the elements we previously mentioned; however, as subjective judgement arises due to the interpretation of information, we may reduce this form of uncertainty to some degree if messages are conveyed in a clear and understandable way. Again, having a good understanding of the knowledge needs and potential purpose of the data allows project managers you to properly shape the communication and thus maximise its effects.

When communicating project findings, care must be taken to not present information in a misleading way, for example, with unfounded certainty (by downplaying the uncertainty) that may undermine project outcomes in the long term (e.g., engagement and empowerment of community members) and produce public distrust. Given that community science projects on alien species may have management implications, which can be highly contentious (Crowley et al. 2019; Friedel et al. 2011; Zengeya et al. 2017), presenting an objective interpretation of research findings will produce the most beneficial outcomes for the project, but also ultimately for public trust in science in general. If the research findings lead to recommendations, it is also important to communicate the level of uncertainty that is relevant for the decision (Fischhoff and Davis 2014), e.g., if uncertainty around a measurement of alien species abundance does not affect the recommendation for its management. Although policies and practices related to the management of alien species are intrinsically value-driven and will therefore strongly influence how messages transmitted are received (Reaser 2001), communicating transparently will establish and maintain trust to the benefit of community science in general. In dealing with uncertainty, communicators also grapple with the issue of credibility. Acknowledging uncertainty and explicitly communicating uncertainty will increase the perceived trustworthiness of the data (Lundgren and McMakin 2018).

## Conclusions

We recognise that community science plays a growing role in the understanding, and ultimately in the prevention and management, of future biological invasions and in the ongoing monitoring of already established alien species (Baker et al. 2019; Dickinson et al. 2012; Eritja et al. 2019). Community science projects provide opportunities to capture information that would otherwise be difficult to record, usually due to high costs and efforts associated with data collection (Brown et al. 2018). They mutually benefit science and society, expanding scientific knowledge and improving science literacy among the general public (Dickinson et al. 2012). However, data generated from community science projects may be associated with varying degrees of uncertainties (Crall et al. 2011; Gardiner et al. 2012; Jiménez et al. 2019; Tye et al. 2017). Community science approaches may be more effective in capturing some uncertainties in natural systems, due to their scale, but may introduce additional measurement error or bias uncertainties due to inconsistent or less skilled observers. Appropriate project design should aim to make the most of the opportunities community science offers and minimise sources of added variability. Neglecting to address these uncertainties, particularly when communicating with participants, stakeholders, managers, and policy- and decision-makers can decrease overall confidence in the results, leading to inappropriate management decisions and public scepticism (van der Bles et al. 2019; Vanderhoeven et al. 2017). Effective uncertainty communication (Box 4) creates a more informed public, empowers community members in the decision-making process and leads to better uptake of management decisions (Vanderhoeven et al. 2017).

**Box 4:** Key messages for effective uncertainty communication

*Uncertainty is an inherent part of the scientific process and will persist to some degree regardless of the approach. The understanding and extent of uncertainty, particularly in relation to our confidence in the results, is critical to open and transparent communication of scientific findings* **Set up your aims:** Establish and communicate a clear objective and indicators of success. **Know your audience:** Define the target audiences, understand their values and motivations, identify their needs and potential agenda, and understand how uncertainty may steer the decision-making process and the decisions. Adapt your message and communication approach accordingly. **Avoid jargon:** Keep the usage of jargon to a minimum and explain scientific terms clearly. **Train participants:** Ensure participants have adequate instructions and understanding so providing benefits through democratisation of science by increasing scientific literacy while also reducing errors and uncertainty. **Develop a reciprocal dialogue:** Communication should occur between project coordinators and volunteer recorders. Channels for easy communication should be set up and encouraged by project organisers. **Ask for feedback:** Actively seek discussion and feedback throughout the communication effort. This will help determine where potential misunderstandings may be arising. **Acknowledge uncertainty and communicate about it explicitly:** Discuss the sources of uncertainty, explain why it exists, describe what, if anything, can be done to manage it better. Explain the level of uncertainty that is relevant for decision-making. **Build trust with your audience:** Be honest, transparent and unbiased in communicating with your audience. Trust between communicator and audience is essential for effective communication. **Share your stories:** We can improve our application of community science to biodiversity studies by highlighting the successes and, importantly, failures of projects while also sharing the excitement of the collaborative outcomes.	

## Supplementary Information

Below is the link to the electronic supplementary material.

Supplementary file 1 (XLSX 53 KB)

## Data Availability

Data are included as Supplementary Material.
